# Supplementary material for: Metabolomics investigation of post-mortem human pericardial fluid
Source: Int J Legal Med. 2023 Jul 4;137(6):1875–85. doi: 10.1007/s00414-023-03050-w (PMC10567860; doi:10.1007/s00414-023-03050-w)
Supplement: Supplementary file 1 — Table S1. List of human PF metabolites quantified using the Chenomx Profiler tool. Figure S1. 1H NMR spectra of (A) UPF and (B) LLEPF samples. Three PF aliquots of the same individual were analyzed. (*) indicates the resonances of ethanol. (DOCX 2745 kb) [file 414_2023_3050_MOESM1_ESM.docx]

Supplementary Material

Metabolomics investigation of post-mortem human pericardial fluid

Alberto Chighine^1,^*, Matteo Stocchero^2^, Giulio Ferino^1^, Fabio De-Giorgio^3,4^, Celeste Conte^3,4^, Matteo Nioi^1^, Ernesto d’Aloja^1^ and Emanuela Locci^1^

^1^ Department of Medical Sciences and Public Health, Section of Legal Medicine, University of Cagliari, Cagliari, Italy.

^2^ Department of Women’s and Children’s Health, University of Padova, Padova, Italy.

^3^ Department of Health Care Surveillance and Bioethics, Section of Legal Medicine, Catholic University of Rome, Rome, Italy.

^4^ Fondazione Policlinico Universitario A. Gemelli, IRCCS, Rome, Italy.

*Corresponding author: Alberto Chighine. E-mail: [alberto.chighine@unica.it](mailto:alberto.chighine@unica.it)

**Table S1.** List of human PF metabolites quantified using the Chenomx Profiler tool.

| **Compound** | **PubChem (CID)** |
| --- | --- |
| 3-Hydroxybutyrate | 92135 |
| Acetate | 176 |
| Acetone | 180 |
| Alanine | 5950 |
| Asparagine | 6267 |
| Aspartate | 5960 |
| Betaine | 248 |
| Butyrate | 264 |
| Choline | 305 |
| Citrate | 311 |
| Creatine | 586 |
| Creatinine | 588 |
| Dimethylamine | 674 |
| Ethanolamine | 700 |
| Formate | 284 |
| Fumarate | 723 |
| Glucose | 5793 |
| Glutamate | 33032 |
| Glutamine | 5961 |
| Glycerol | 753 |
| Glycine | 750 |
| Histidine | 6274 |
| Hypoxanthine | 790 |
| Inosine | 6021 |
| Isoleucine | 6306 |
| Lactate | 108689 |
| Leucine | 6106 |
| Lysine | 5962 |
| Maltose | 439186 |
| Mannose | 18950 |
| Methanol | 887 |
| Methionine | 6137 |
| Nicotinurate | 68499 |
| Ornithine | 6262 |
| Phenylalanine | 6140 |
| Proline | 145742 |
| Propionate | 1032 |
| Serine | 5951 |
| Succinate | 1110 |
| Taurine | 1123 |
| Threonine | 6288 |
| Trimethylamine | 1146 |
| Tryptophan | 6305 |
| Tyrosine | 6057 |
| Uracil | 1174 |
| Uridine | 6029 |
| Valine | 6287 |
| myo-Inositol | 892 |
| sn-Glycero-3-phosphocholine | 439285 |
| β-Alanine | 239 |

**Fig. S1**. ^1^H NMR spectra of (A) UPF and (B) LLEPF samples. Three PF aliquots of the same individual were analysed. (*) indicates the resonances of ethanol.


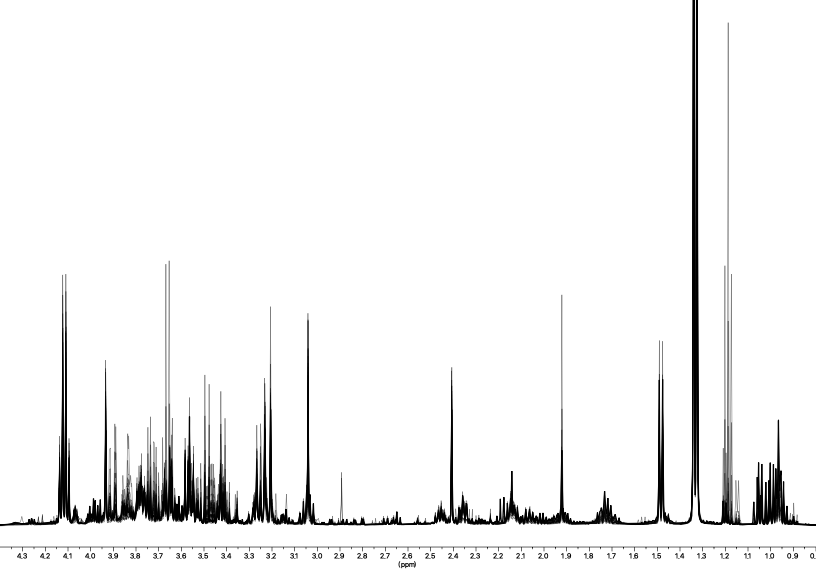

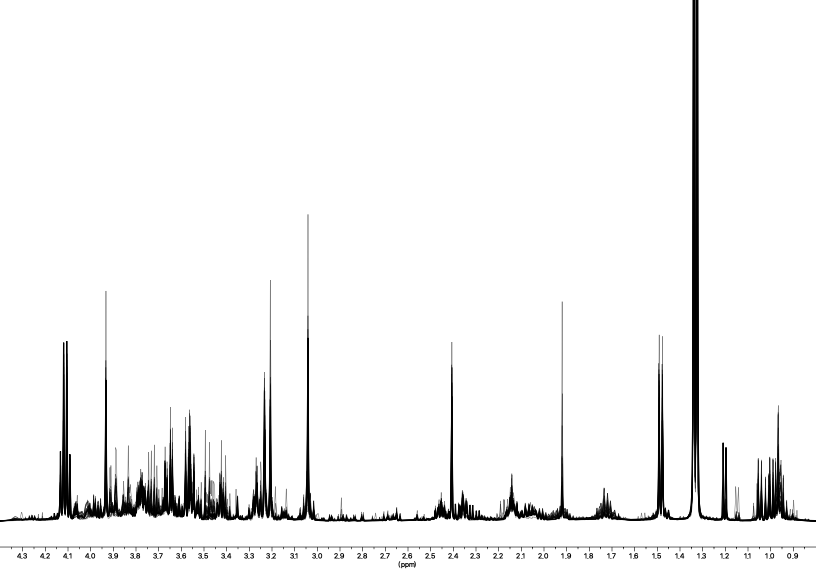


*****

*****

**B**

**A**
